# A Moderate Zinc Deficiency Does Not Alter Lipid and Fatty Acid Composition in the Liver of Weanling Rats Fed Diets Rich in Cocoa Butter or Safflower Oil

**DOI:** 10.1155/2017/4798963

**Published:** 2017-03-29

**Authors:** Edgar Weigand, Jennifer Egenolf

**Affiliations:** Institute of Nutritional Physiology and Animal Nutrition, Justus Liebig University, Heinrich-Buff-Ring 29-32, 35392 Giessen, Germany

## Abstract

The aim of the study was to examine whether a moderate zinc deficiency alters hepatic lipid composition. Male weanling rats, assigned to five groups (8 animals each), were fed low-carbohydrate high-fat diets supplemented with 7 or 50 mg Zn/kg (LZ or HZ) and 22% cocoa butter (CB) or 22% safflower oil (SF) for four weeks. One group each had free access to the LZ-CB and LZ-SF diets, one group each was restrictedly fed the HZ-CB and HZ-SF diets in matching amounts, and one group had free access to the HZ-SF diet (ad libitum control). The rats fed the LZ diets had significantly lower energy intakes and final body weights than the ad libitum control group, and lower plasma and femur Zn concentrations than the animals consuming the HZ diets. Hepatic cholesterol, triacylglycerol and phospholipid concentrations, and fatty acid composition of hepatic triacylglycerols and phospholipids did not significantly differ between the LZ and their respective HZ groups, but were greatly affected by dietary fat source. In conclusion, the moderate Zn deficiency did not significantly alter liver lipid concentrations and fatty acid composition.

## 1. Introduction

Zinc (Zn) is the second most abundant trace element in the human and animal body. It is an essential cofactor for many hundreds of enzymes and numerous other proteins that fulfill a wide variety of biochemical processes in metabolism [1, 2]. Poor Zn status is considered to be one of the most common micronutrient deficiencies in human populations worldwide [3, 4]. Zn deficiency has been associated with many diseases, including diabetes, chronic liver disease, and cardiovascular disease [5]. Zn supplementation showed beneficial effects on plasma lipid parameters and may have the potential to reduce the incidence of atherosclerosis [5]. The impact of Zn deficiency on lipid metabolism has been studied extensively using rodents as models. Young animals can be readily depleted of zinc due to their high nutritional demands for growth, unlike adult humans [6]. Since Zn depletion of young animals causes anorexia and growth retardation, classical designs included limit-fed control animals in order to account for metabolic effects of reduced energy intake. Dietary energy restriction has been shown to effect significant changes in hepatic lipid composition under conditions of adequate Zn nutrition [7]. An alterative experimental paradigm widely used in Zn studies has been force-feeding young rats by gastric tube in order to equalize and synchronize food intake [8–11]. In these studies, Zn-depleted animals generally developed fatty livers and an altered fatty acid (FA) composition of liver lipids. In contrast, livers of rats given free access to Zn-deficient diets did not display increased triacylglycerol (TAG) concentrations compared with those of Zn-adequate controls [12–15]. Most former studies are based on models of severe Zn deprivation. Marginal Zn deficiency is the more prevalent phenotype among human populations than clinical states of Zn depletion [5, 6].

The aim of our study was to investigate the effect of a moderate Zn depletion on hepatic lipid and FA composition in weanling rats fed diets rich in cocoa butter or safflower oil as sources of saturated and polyunsaturated FAs, respectively. Dietary fat source has been reported to interact with the effect of dietary Zn depletion on FA composition of hepatic lipids in young rats [9, 10, 15]. High-fat diets are apt to foster FA oxidation for maintenance and growth [16]. A mild Zn deficit allows a significant accretion of lean tissue including membrane lipids during the growth spurt in the postweaning period, whereas severe Zn depletion leads to growth arrest by impairing cell division and proliferation [2].

## 2. Methods

### 2.1. Animals and Experimental Design

A total of 40 male weanling Wistar rats (Harlan-Winkelmann, Borchen, Germany) with an initial body weight of 50.8 ± 0.2 g (mean ± SD) were divided into five groups of eight animals each. These groups were randomly assigned to one of four semisynthetic diets that were supplemented with 7.0 or 50 mg zinc as Zn sulfate per kg (LZ and HZ diets, resp.) and with either cocoa butter (CB) or safflower oil (SF). Dietary treatments of the groups were as follows: (1) LZ-CB, fed the LZ-CB diet free choice, (2) HZ-CBR, fed the HZ-CB diet in restricted amounts equal to intake in the LZ-CB group on the previous day, (3) LZ-SF, fed the LZ-SF diet free choice, (4) HZ-SFR, fed the HZ-SF diet in restricted amounts equal to intake in the LZ-SF group on the previous day, and (5) HZ-SF, fed the HZ-SF diet free choice (ad libitum control). An ad libitum-fed HZ-CB control group was not included because dietary fat source did not affect food intake and growth of weanling rats fed Zn-adequate diets free choice in our former experiment [15]. All animals had free access to demineralized water. They were kept individually in metabolic cages (stainless-steel metal grids) under controlled environmental conditions (22°C, 60% rel. humidity, 12 h dark-light cycle). Food remainders were removed daily and weighed. During wk 3 and 4, faeces were collected quantitatively from each animal and stored at −20°C until analysis. After four weeks, food was withdrawn overnight for 10 to 12 h before the animals were anesthetized in a carbon dioxide atmosphere and killed by decapitation. Blood was collected in heparinized tubes to prepare plasma by centrifugation (10 min at 1500 ×g). The liver and right femur bone were removed from the carcasses and stored at −80°C. All experimental treatments of the rats followed established guidelines for the care and handling of laboratory animals. Approval was obtained by the Animal Protection Authority of the State (II 25.3-19c20/15c GI 19/3).

### 2.2. Diets

**Figure 1:**
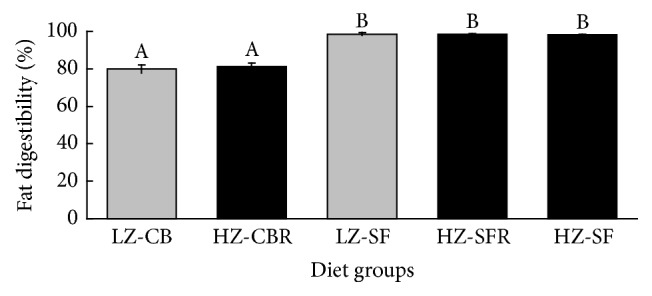
Fat digestibility during wk 3 and 4 of feeding male weanling rats diets containing 22% cocoa butter or safflower oil. Columns represent means ± SDs, *n* = 8. Labeled means without a common letter differ, *P* < 0.05 (1-factor ANOVA followed by Tukey's multiple-comparison test). Diet groups: LZ-CB, low-Zn (LZ) cocoa butter (CB) diet fed free choice; HZ-CBR, high-Zn (HZ) CB diet fed in restricted amounts equivalent to intake of the LZ-CB diet; LZ-SF, LZ safflower oil (SF) diet fed free choice; HZ-SFR, HZ-SF diet fed in restricted amounts equivalent to intake of the LZ-SF diet; HZ-SF, HZ-SF diet fed free choice.

All diets contained, per kg, 200 g powdered egg albumen, 67 g corn starch, 100 g sucrose, 280 g cellulose, 30 g soybean oil, 3.0 g lysine plus methionine (1 : 1 by wt.), 100 g mineral plus vitamin premixes [17], and 220 g cocoa butter or 220 g safflower oil. They were stored at 4°C after preparation. The high cellulose addition served to restrict the dietary energy density. Except for the fat components, the dietary metabolizable (ME) contents are based on tabulated values of the ingredients [18]. Fat digestibility of the CB and SF diets, assessed during wk 3 and 4, significantly differed (*P* < 0.001) (Figure 1) and averaged (overall mean ± SD) 80.5 ± 2.0 (*n* = 16) and 98.4 ± 0.6% (*n* = 24), respectively. On the basis of 39 kJ/g digestible fat, the CB and SF diets were estimated to contain 15.1 and 16.9 kJ ME per gram dry matter, respectively. Carbohydrates (starch plus sucrose) and fat contributed 24.3 and 55.0% of the ME in the CB diets, and 21.7 and 59.9% in the SF diets. The LZ-CB, LZ-SF, HZ-CB, and HZ-SF diets contained, by analysis (mean ± SD, *n* = 5), 7.8 ± 0.6, 7.8 ± 0.7, 53 ± 7.3, and 53 ± 6.4 *μ*g Zn/g dry matter, respectively. Based on energy density, Zn concentrations in the LZ-CB and LZ-SF diets were 0.517 and 0.462 *μ*g/kJ ME, respectively.  Table 1 presents the analytical FA composition of the diets.

### 2.3. Analytical Methods

#### 2.3.1. Zn Analyses

Plasma samples were diluted with 0.1 M HCl and analyzed for zinc by atomic absorption spectrometry (PU 9400, Phillips, Kassel, Germany). Samples of diets, livers, and femur bones were wet-ashed with 65% (w/vol) HNO_3_ for 16 h, and appropriately diluted with aqua bidest for Zn analysis by ICP-AES (Type 701, Unicam). Zn analyses were replicated at least twice per sample, and accuracy was validated by the analysis of standard samples of known Zn concentration.

#### 2.3.2. Lipid Analyses

The fat content of diets and faeces (collected in wk 3 and 4) was analyzed by an official method [19] for the determination of fat digestibility. Faeces were treated with 4 M HCl before fat extraction.

Liver concentrations of total lipids, cholesterol, TAGs, and phospholipids (PLs) were determined in duplicate lipid extracts as previously described [15]. Precision and accuracy of cholesterol and TAG assays were assessed with Qualitrol (Merck, Darmstadt, Germany).

#### 2.3.3. FA Analyses

Diet samples were heated in 0.4 M HCl and dried before lipid extraction by n-hexane (0.005% BHT). Liver PLs and TAGs were isolated from lipid extracts [15] by solid-phase extraction [20]. Briefly, hepatic lipid extracts were vacuum-dried, redissolved in chloroform-isopropanol (C-I, 2 : 1, by vol), and fractionated with hexane-conditioned aminopropyl Bond Elut columns (Bond Elut NH2 500, Agilent Technologies). Neutral lipids were eluted with C-I, free FAs with 2% acetic acid (by vol) in diethylether, and the polar PLs with methanol. The neutral lipid fraction was redissolved in n-hexane after removal of the C-I eluant and transferred to fresh Bond Elut columns to elute cholesteryl esters with n-hexane and finally to elute TAGs with n-hexane containing 1% diethylether and 10% dichloromethane (by vol). PL and TAG fractions were vacuum-dried, redissolved in isopropanol (0.005% BHT), and stored at −80°C for FA analysis.

Lipid extracts of diets and liver PLs and TAGs fractions were supplemented with 1,2,3-triheptadecanoylglycerol as internal standard, condensed at 45°C under a nitrogen stream, redissolved in n-hexane (0.005% BHT), and transmethylated with N-trimethylsulfoniumhydroxide (Macherey & Nagel) at room temperature [21]. The FA methylesters (FAMEs) were separated and quantified by a GLC system (Chrompack 9400) that was equipped with an autosampler, a 50 m Permabond FFAP-DF column (Macherey & Nagel), and a flame ionization detector. Hydrogen was used as carrier gas. FAMEs were separated in a temperature gradient program and identified on the basis of their retention times compared to an authentic FAME mix (C4–C24, number 18919-1AMP Supelco, Sigma-Aldrich) that was supplemented with three additional polyenoic FAs (*cis*-13,16,19-docosatrienoic acid, Sigma;* cis*-7,10,13,16-docosatetraenoic acid, Sigma;* cis*-7,10,13,16,19-docosapentaenoic acid, Supelco). FAME peak areas were quantified in relation to the peak areas of the internal standard (heptadecanoic acid). The relative unsaturation index (UI) of FAs in diets, liver TAGs, and PLs was calculated by multiplying the molar percentages of FAs by the number of double bonds present and dividing the total sum of products by hundred [22].

### 2.4. Statistical Analyses

The data of the five diet groups were statistically analyzed by using SPSS for Windows (version 19; IBM). Homogeneity of variance was checked by Levene's test. In the case of heterogeneity of variance, data were logarithmically transformed. Data of the five groups were subjected to one-factor ANOVA, followed by Tukey's multiple-comparison test (the level of significance being set at *P* < 0.05). In addition, results of the LZ-CB, HZ-CBR, LZ-SF, and HZ-SFR groups were analyzed by bifactorial ANOVA to test for main effects of Zn, fat (fat source), and Zn × fat interaction. Correlations are based on Pearson's correlation coefficients.

## 3. Results

### 3.1. Food and Energy Intake, Final Body Weights, and Zn Status of the Rats

Food and ME intake and final body weights of the weanling rats fed the LZ-CB and LZ-SF diets free choice were comparable to those of the rats fed the corresponding HZ diets in equivalent amounts but markedly lower than those recorded for the animals fed the HZ-SF diet free choice (Table 2). Dietary fat source also significantly affected food and ME intake and final body weights, which were at least 20% lower in the LZ-SF than in the LZ-CB group. ME intake per gram of body weight gain was similar among groups except for a significantly (*P* < 0.05) higher value in the case of the LZ-SF group. Zn intake per gram body weight gain did not differ between the LZ-CB and LZ-SF groups (*P* > 0.05), but was higher (*P* < 0.05) in the HZ-CBR group than in the HZ-SFR and -SF groups.

**Figure 2:**
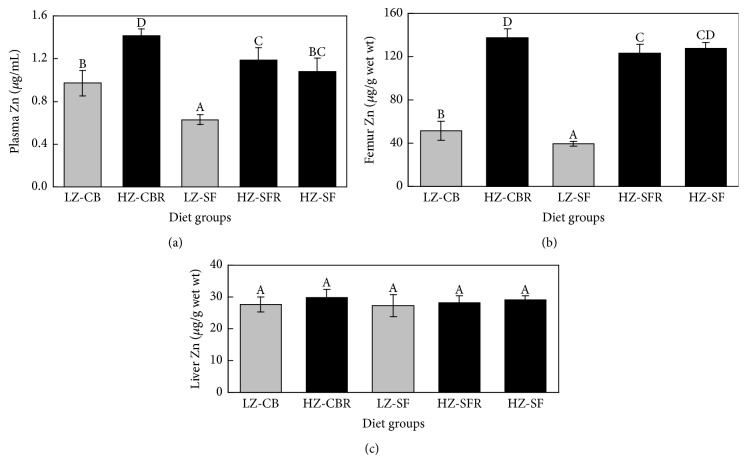
Plasma, femur, and liver Zn concentrations of male weanling rats fed high-fat diets differing in Zn content and fat source for 4 wk. Columns represent means ± SDs, *n* = 8. Labeled means without a common letter differ, *P* < 0.05 (1-factor ANOVA followed by Tukey's multiple-comparison test). Bifactorial ANOVA of the LZ-CB, HZ-CBR, LZ-SF, and HZ-SFR: significance (*P*) of Zn, fat, and zinc × fat is <0.001, <0.001, and 0.098 for plasma Zn, <0.001, 0.006, and 0.82 for femur Zn, and 0.13, 0.32, and 0.54 for liver Zn concentrations, respectively. Diet groups: LZ-CB, low-Zn (LZ) cocoa butter (CB) diet fed free choice; HZ-CBR, high-Zn (HZ) CB diet fed in restricted amounts equivalent to intake of the LZ-CB diet; LZ-SF, LZ safflower oil (SF) diet fed free choice; HZ-SFR, HZ-SF diet fed in restricted amounts equivalent to intake of the LZ-SF diet; HZ-SF, HZ-SF diet fed free choice.

Plasma and femur Zn concentrations were greatly reduced in the LZ-CB and LZ-SF groups compared with the HZ groups (Figure 2). Furthermore, the rats fed the LZ- and HZ-SF diets had lower (*P* < 0.05) plasma and femur Zn concentrations than the animals fed the corresponding LZ- and HZ-CB diets. In contrast, liver Zn concentrations were not affected by dietary treatments (*P* > 0.05).

### 3.2. Liver Lipid Concentrations

The dietary Zn level did not significantly alter hepatic concentrations of total lipids, cholesterol, TAGs, and PLs (Table 3). But livers of the rats fed the SF diets had significantly (*P* < 0.05) higher concentrations of cholesterol and TAGs than those consuming the CB diets. The highest total lipid and TAG concentrations were recorded in the rats fed the HZ-SF diet free choice. CB-fed rats had approximately 10% higher PL concentrations than the SF-fed animals (*P* < 0.05).

### 3.3. FA Composition of Liver PLs

The bifactorial ANOVA of the FA composition of hepatic PLs displays significant (*P* < 0.05) Zn effects in the case of palmitic acid (16:0), dihomo-*γ*-linolenic acid (20:3n-6), arachidonic acid (20:4n-6), total n-6 polyunsaturated FAs (n-6 PUFAs), and the ratios n-6/n-3 PUFAs (Table 4). These effects mainly result from differences between the LZ-SF and HZ-SFR groups. Dietary fat source markedly affected the proportions of all FAs except for palmitic acid. Saturated FAs (SFAs) accounted for 43 and 40 mol%, and n-6 PUFAs for about 40 and 50% of the total FAs in the CB and SF groups, respectively. In all five diet groups, arachidonic acid was the most abundant PUFA of PLs and contributed at least one-third of the total FAs. Molar proportions of n-3 PUFAs were approximately threefold higher than the proportions of monounsaturated FAs (MUFAs). Docosahexaenoic acid (22:6n-3) was the prevailing n-3 PUFA and present in significantly (*P* < 0.05) greater amounts in the CB than in the SF groups. Total Δ^6^ desaturation products of linoleic (18:2n-6) and *α*-linolenic acid (18:3n-3) account for approximately 90% of the total PUFAs in the CB-fed rats and for 80% in the SF-fed animals. The ratios of n-6/n-3 PUFAs averaged 3.2 in the two CB groups, whereas they were approximately twice as high in the three SF groups. The relative UI of the liver PLs, however, was closely comparable among the five diet groups (*P* = 0.32).

### 3.4. FA Composition of Liver TAGs

The FA composition of liver TAGs shows significant (*P* < 0.05) Zn effects on the molar proportions of SFAs, largely because of differences between the LZ-CB and HZ-CBR groups (Table 5), which accordingly affect the relative UI. Proportions of MUFAs and PUFAs were not significantly (*P* > 0.05) altered by the dietary Zn level. TAGs of the HZ-SFR and HZ-SF groups contained closely comparable proportions of total SFAs, MUFAs, and PUFAs. Dietary fat source, however, greatly modified the FA pattern. The abundance of SFAs in the CB groups was nearly three times as high as in the SF groups (*P* < 0.001). Oleic acid (18:1n-9) was the prevailing single FA in TAGs of the CB-fed rats, whereas linoleic acid dominated in TAGs of the SF-fed animals. The proportions of n-3 PUFAs did not exceed 2.5 and 1.5 mol% in the CB- and SF-fed animals, respectively. The ratios of n-6/n-3 PUFAs in the SF groups were approximately eightfold and the relative UI twofold higher than in the CB groups.

### 3.5. Correlations of Liver FA Composition

The total proportions of SFAs, MUFAs, n-6-PUFAs, and n-3 PUFAs in liver TAGs of the two CB groups and the three SF groups significantly correlate with their molar abundance in the CB (*r* = 0.80, *P* < 0.02, and *n* = 8) and SF diets (*r* = 0.99, *P* < 0.001, and *n* = 12), respectively. In the case of the PLs, the corresponding correlations are much lower (*r* = 0.52, *P* < 0.20 in the CB-fed rats and *r* = 0.71, *P* < 0.01 in the SF-fed rats).

## 4. Discussion

Dietary Zn deficiency is well known to depress appetite and growth of young animals. In the current study, food intake and final body weights of the rats fed the LZ-CB and -SF diets were markedly reduced compared to values of the animals freely consuming the HZ-SF diet (Table 2). Remarkably, energy intake and final body weights of the rats on the LZ-SF diet were 20% lower than those of the animals on the LZ-CB diet. Plasma and femur Zn concentrations indicate a greater degree of Zn depletion of the LZ-SF rats (Figure 2). Due to a major difference in fat digestibility (Figure 1), Zn concentration of the LZ-CB diet was nearly 12% higher than that of the LZ-SF diet based on energy density (0.517 versus 0.462 *μ*g Zn/kJ ME, resp.). Such a difference in Zn concentration can be expected to affect the growth response. However, other studies also suggest that Zn-deficient diets rich in PUFA may worsen appetite, growth, and Zn status of young rats [15, 23, 24] and chicks [25].

Many studies, largely conducted on rodent models, suggest that Zn deficiency may lead to profound changes in hepatic lipid composition (reviewed in [26]). In our experiment, the moderate Zn depletion of the rats fed the LZ diets did not significantly alter the concentrations of hepatic lipids compared to those of the animals fed the corresponding HZ diets in equivalent amounts (Table 3). The two-thirds higher TAG levels in the liver of the rats given free access to the HZ-SF diet than those of the animals fed this diet in restricted amounts or freely fed the LZ-SF diet may be attributed to the higher energy intake in the HZ-SF group. This notion is consistent with former studies showing that hepatic TAG concentrations in Zn-deficient rats were lower than in control animals given free access to Zn-adequate diets [12–15, 23] but comparable to TAG levels in restrictedly fed control animals [15, 27, 28]. In contrast, research in which young rats were fed by gastric tube in order to equalize and synchronize food intake consistently found that Zn depletion caused greatly increased liver TAG concentrations compared to levels of control animals [8–11], unless diets contained oils rich in n-3 PUFAs [26]. Force-feeding young rats severely Zn-deficient diets in excess of voluntary consumption induces overfeeding, which can be expected to downregulate the hepatic expression of genes involved in fat catabolism [11], stimulate lipogenic pathways instead [11, 29], and hence provoke fatty livers. Zn deprivation impairs the accretion of lean tissue, including protein and membrane lipids. Nonalcoholic fatty liver disease, which has become worldwide the most prevalent chronic liver disease in humans [30], is primarily attributed to long-term overeating and physical inactivity and not to a deficit of zinc as the leading cause, although subclinical Zn deficiency is considered a widespread phenotype among human populations [3–5] and Zn supplementation has shown beneficial effects, especially on diabetic patients [31]. An aberrant hepatic fat accumulation was not among the changes observed in model studies of experimental mild Zn deficiency in human volunteers [32].

Independent of the dietary Zn level, the SF-fed rats had appreciably higher hepatic cholesterol and TAG concentrations than those consuming the CB diets. This is consistent with many studies finding higher cholesterol and TAG concentrations in the liver of rats fed diets rich in unsaturated fats compared with saturated fats [15, 33–35]. Some studies point to a lower lipoprotein secretion in response to the ingestion of PUFAs relative to SFAs [34, 36]. In the present experiment, cocoa butter as fat source may have contributed to the marked difference in hepatic cholesterol concentrations between the CB and SF diet groups. Its ingestion has been reported to reduce the intestinal reabsorption of cholesterol [37] and increase faecal loss of bile acids [35].

Former research has shown that diets highly enriched with fats suppress hepatic de novo FA synthesis [15, 38, 39]. Thus, with fat being the major energy source in the present experiment (55 to 60% of the energy intake), it is reasonable to assume that body FAs were essentially of dietary origin, with alterations resulting from ß-oxidation and chain modifications. Numerous animal studies found that Zn deficiency per se does not impede ß-oxidation of FAs, including unsaturated FAs (reviewed in [40]). Likewise, there is ample evidence that Zn depletion does not hinder chain desaturation and elongation of linoleic acid and *α*-linolenic acid (reviewed in [41]). In our experiment, the bifactorial ANOVA displays significant “Zn” effects on the proportions of dihomo-*γ*-linolenic and arachidonic acid in liver PLs, being lower in the LZ groups than in the restrictedly fed HZ groups (Table 4). Overall, however, our data suggest that these differences in PUFA composition are not Zn-specific but instead due to an adaptive response to the limited food allocation in the HZ-CBR and -SFR groups. Firstly, the proportions of n-6 metabolites, and palmitic acid as well, in the PLs of the rats fed the LZ-SF diet deviated less from those fed the HZ-SF diet ad libitum than from those fed this diet restrictedly. Secondly, proportions of total Δ^6^ desaturation products in both liver PLs and TAGs did not differ between the LZ and their corresponding restrictedly fed HZ groups, indicating that the deficit of zinc did not impede desaturation and elongation of linoleic and *α*-linolenic acid. This is in line with former studies conducted under conditions of moderate and severe Zn deficiency in young rats fed low- and high-fat diets [15, 42, 43].

Hepatic PLs in the LZ-SF and HZ-SFR groups, compared to those in the ad libitum-fed HZ-SF group, contained significantly more linoleic acid but less docosahexaenoic acid. Such differences in PUFA composition of liver PLs were observed previously between Zn-deficient and Zn-adequate rats given free access to diets containing vegetable oils rich in linoleic acid [14, 15, 42, 43]. However, linoleic acid proportions of Zn-deficient rats resembled those of pair-fed control animals [42, 43]. Dietary energy restriction has also been found to increase linoleic acid levels in liver TAGs, suggesting that a reduced energy intake mediates a selective retention of linoleic acid [44]. In our experiment, linoleic acid proportions in liver TAGs did not significantly differ among the SF-fed rats. Considering the differences in TAG concentrations, the TAG-associated linoleic acid pool of the rats fed the HZ-SF diet ad libitum was 2.1- and 1.7-fold higher than those of the LZ-SF and HZ-SFR groups, respectively. These differences suggest that more linoleic acid could be incorporated into a substantially greater hepatic TAG pool, thus favouring a higher incorporation of docosahexaenoic acid into PLs of the rats fed the HZ-SF diet free choice. Cunnane [14] had previously pointed out the importance of pool size of neutral lipids in experiments investigating the impact of Zn nutrition on proportions of essential FAs and their longer chain metabolites in liver PLs. Remarkably, studies using the force-feeding protocol found significantly less linoleic acid in liver PLs of Zn-depleted rats than in control animals [9, 45]. This conflicting finding is nevertheless consistent with our results and those of other studies [15, 42, 43] when realizing that hepatic TAG concentrations in force-fed Zn-deficient rats exceeded those of Zn-adequate controls as discussed before.

Oleic acid can originate either from dietary intake or from Δ^9^ desaturation of stearic acid. In our experiment, MUFA proportions, mainly oleic acid, in both liver PLs and TAGs, were comparable between the LZ groups and their corresponding restrictedly fed HZ groups, indicating that dietary Zn deficiency did not affect MUFA incorporation. The nutritional conditions in our experiment are not suitable to question whether a moderate Zn deficit may affect the Δ^9^ desaturation pathway. For the CB-fed rats, there may have been no real need for Δ^9^ desaturation of stearic acid, because their diet was a rich source of oleic acid. For the SF-fed animals, for which the dietary supply of stearic and oleic acid was appreciably lower, it may be assumed that little if any oleic acid was newly synthesized, because PUFAs suppress the expression and activity of the hepatic stearoyl-CoA desaturase [44].

The dietary fat sources used in our experiment, cocoa butter versus safflower oil, exerted prominent effects on the FA composition of both liver PLs and TAGs. The impact on the FA pattern of PLs, however, was much less pronounced than that on TAGs. Evidently, FA composition of PLs is controlled within close limits in view of the important roles that these lipids play as essential structural and functional components of cells. Notably, the relative UI of the hepatic PLs remained closely comparable among the five groups despite major differences in the proportions of unsaturated FAs between the CB- and SF-consuming rats. The markedly lower abundance of docosahexaenoic acid in the hepatic glycerides of the SF rats compared to those of the CB animals may be ascribed to the known competition among linoleic and *α*-linolenic acid for Δ^6^ desaturation in the pathway of producing very-long-chain PUFAs [46].

Taken together, our data indicate that a difference in energy intake due to restricted versus ad libitum feeding is more apt to alter hepatic lipid composition than a moderate Zn depletion. It may be argued that a greater degree of Zn deprivation would be more likely to evoke changes along lipid pathways that are clearly specific for zinc rather than metabolic adaptations to an altered energy status. Liver Zn concentrations of severely Zn-depleted young rats that caused near growth arrest were no lower than approximately 10% of those recorded in our experiment [47, 48] or no lower than those of Zn-adequate control animals [27, 49]. Choosing an experimental model of an increased Zn deprivation that ultimately leads to growth arrest of young animals inevitably lowers nutritional demands towards the maintenance level and suppresses the accretion of lean tissue, including cellular membrane lipids, while zinc remains the first limiting nutrient and not energy or any other essential nutrient, whereas control animals fed the Zn-supplemented control diet in equivalent amounts are forced to adapt to a metabolic state of severe energy shortage. In our experiment, the LZ rats and their limit-fed HZ controls more than tripled their body weights during the 4-week period.

## 5. Conclusions

The moderate dietary Zn deficiency markedly lowered the Zn status of the young rats as indicated by reduced final body weights and plasma and femur Zn concentrations. There was, however, no evidence that the dietary shortage of zinc as such altered hepatic lipid concentrations and FA composition of PLs and TAGs, including long-chain metabolites of linoleic and *α*-linolenic acid. Seemingly Zn-related changes in the FA composition of these glycerolipids could be attributed to the restricted feeding of the Zn-sufficient animals. Our study suggests that hepatic lipid metabolism is rather unresponsive to a nutritional Zn deficit in contrast to its response to differences in energy status imposed by the feeding protocol assigned to controls and the pronounced impact of the dietary fat source.

## Figures and Tables

**Table 1:** Fatty acid composition (mol/100 mol) of the experimental diets fed to weanling rats for 4 wk.

Fatty acids	CB diets	SF diets
Palmitic (16:0)	26.3	7.9
Palmitoleic (16:1)	0.2	0.1
Stearic (18:0)	29.0	2.3
Oleic (18:1n-9)	31.6	12.5
Linoleic (18:2n-6)	10.5	75.3
*α*-Linolenic (18:3n-3)	1.2	1.1
SFAs^1^	56.4	10.7
MUFAs^2^	31.9	12.9
PUFAs	11.7	76.4
PUFA/SFA ratio	0.21	7.14
n-6/n-3 ratio	8.8	68.5
Relative UI	0.57	1.67

^1^Contain very long-chain SFAs.

^2^Contain very long-chain MUFAs.

SFAs, saturated fatty acids; MUFAs, monounsaturated fatty acids; PUFAs, polyunsaturated fatty acids; UI, unsaturation index.

**Table 2:** Food and ME Zn intake and final body weights of male weanling rats fed high-fat diets differing in Zn content and fat source free choice or in restricted amounts for 4 wk.

		Diet groups^1,2^					2 × 2 ANOVA^3^: *P* level		
		LZ-CB	HZ-CBR	LZ-SF	HZ-SFR	HZ-SF	Zn	Fat	Zn × fat
Food intake	g DM/d	13.2^b^ ± 0.9	12.9^b^ ± 0.4	9.4^a^ ± 1.0	9.4^a^ ± 0.2	14.5^c^ ± 0.4	*0.70*	*<0.001*	*0.44*
ME intake	kJ/d	199^b^ ± 14.1	195^b^ ± 6.5	159^a^ ± 16.9	160^a^ ± 2.3	245^c^ ± 7.3	*0.73*	*<0.001*	*0.45*
ME intake	kJ/g BWG	36.3^a^ ± 1.8	35.6^a^ ± 1.7	39.4^b^ ± 2.1	36.1^a^ ± 1.6	36.7^a^ ± 1.1	*0.005*	*<0.001*	*0.052*
Zn intake^4^	*μ*g/g BWG	18.7^a^ ± 0.9	124.9^c^ ± 6.1	18.2^a^ ± 1.0	113.2^b^ ± 4.9	115.2^b^ ± 3.6	*<0.001*	*0.001*	*0.062*
Final body weights	g	205^b^ ± 14.8	205^b^ ± 11.5	164^a^ ± 18.2	175^a^ ± 9.9	238^c^ ± 13.7	*0.31*	*<0.001*	*0.21*

^1^LZ-CB, low-Zn (7.8 *μ*g Zn/g DM) cocoa butter (CB) diet fed free choice; HZ-CBR, high-Zn (53 *μ*g Zn/g DM) CB diet fed in restricted amounts equivalent to the intake of the LZ-CB diet; LZ-SF, low-Zn (7.8 *μ*g Zn/g DM) safflower oil (SF) diet fed free choice; HZ-SFR, high-Zn (53 *μ*g Zn/g DM) SF diet fed in restricted amounts equivalent to the intake of the LZ-SF diet; HZ-SF, HZ-SF diet fed free choice.

^2^Values are means ± SD, *n* = 8. Labeled means in a row without a common letter differ by 1-factor ANOVA followed by Tukey's multiple-comparison test, *P* < 0.05.

^3^Bifactorial ANOVA of the LZ-CB, HZ-CBR, LZ-SF, and HZ-SFR diet groups.

^4^1-factor and bifactorial ANOVA after logarithmic transformation of the data.

BWG, body weight gain; DM, dry matter; ME, metabolizable energy.

**Table 3:** Lipid concentrations in the liver (wet weight) of male weanling rats fed high-fat diets differing in Zn content and fat source free choice or in restricted amounts for 4 wk.

		Diet groups^1,2^					2 × 2 ANOVA^3^: *P *level		
		LZ-CB	HZ-CBR	LZ-SF	HZ-SFR	HZ-SF	Zn	Fat	Zn × fat
Total lipids	mg/g	53.3^a^ ± 6.0	57.2^a^ ± 9.5	66.2^a^ ± 14.5	69.3^a^ ± 6.3	95.8^b^ ± 17.8	*0.16*	*0.003*	*0.77*
Cholesterol^4,5^	*μ*mol/g	5.8^a^ ± 0.7	5.2^a^ ± 0.6	10.6^b^ ± 2.0	9.6^b^ ± 1.1	10.3^b^ ± 2.6	*0.08*	*<0.001*	*0.86*
TAGs^4^	*μ*mol/g	20.4^a^ ± 5.7	21.0^a^ ± 6.7	30.0^b^ ± 10.7	35.6^b^ ± 6.1	59.6^c^ ± 23.6	*0.25*	*<0.001*	*0.37*
PLs	*μ*mol/g	42.8^ab^ ± 2.6	43.1^b^ ± 4.2	39.1^a^ ± 2.6	38.8^a^ ± 2.1	39.0^ab^ ± 2.3	*0.99*	*0.001*	*0.81*

^1^See footnote 1 of Table 2.

^2^Values are means ± SD, *n* = 8. Labeled means in a row without a common letter differ by 1-factor ANOVA followed by Tukey's multiple-comparison test, *P* < 0.05.

^3^Bifactorial ANOVA of the LZ-CB, HZ-CBR, LZ-SF, and HZ-SFR diet groups.

^4^1-factor ANOVA after logarithmic transformation of the data.

^5^Bifactorial ANOVA after logarithmic transformation of the data.

TAGs, triacylglycerols; PLs, phospholipids.

**Table 4:** Fatty acid composition (mol/100 mol) of liver phospholipids in male weanling rats fed high-fat diets differing in Zn content and fat source free choice or in restricted amounts for 4 wk.

Fatty acids	Diet groups^1,2^					2 × 2 ANOVA^3^: *P* level		
	LZ-CB	HZ-CBR	LZ-SF	HZ-SFR	HZ-SF	Zn	Fat	Zn × fat
16:0	15.7^a^ ± 0.55	14.9^a^ ± 1.15	16.0^ab^ ± 0.52	14.9^a^ ± 0.77	16.3^b^ ± 0.66	*0.003*	*0.59*	*0.56*
18:0	27.5^b^ ± 0.89	28.3^b^ ± 0.90	24.2^a^ ± 1.39	23.7^a^ ± 1.71	25.4^a^ ± 1.09	*0.85*	*<0.001*	*0.14*
Σ **SFAs**	43.2^c^ ± 0.58	43.2^c^ ± 1.23	40.2^ab^ ± 1.73	38.6^a^ ± 2.04	41.7^bc^ ± 1.63	***0.14***	***<0.001***	***0.12***
18:1n-9	4.0^b^ ± 0.43	3.7^b^ ± 0.35	1.9^a^ ± 0.30	2.2^a^ ± 0.23	1.8^a^ ± 0.20	*0.99*	*<0.001*	*0.056*
Σ **MUFAs**^4^	4.5^b^ ± 0.62	4.1^b^ ± 0.42	2.5^a^ ± 0.36	2.7^a^ ± 0.26	2.3^a^ ± 0.23	***0.59***	***<0.001***	***0.10***
18:2n-6	4.8^a^ ± 1.17	4.5^a^ ± 1.10	10.7^c^ ± 1.03	10.6^c^ ± 0.81	7.4^b^ ± 0.90	*0.53*	*<0.001*	*0.84*
20:3n-6	0.4^a^ ± 0.14	0.4^ab^ ± 0.07	0.3^a^ ± 0.05	0.5^b^ ± 0.10	0.3^a^ ± 0.07	*0.001*	*0.52*	*0.050*
20:4n-6	33.8^a^ ± 0.67	34.6^ab^ ± 1.13	35.7^bc^ ± 1.45	37.3^c^ ± 1.66	36.7^c^ ± 0.92	*0.012*	*<0.001*	*0.40*
22:4n-6^5^	0.5^a^ ± 0.09	0.5^a^ ± 0.06	1.6^b^ ± 0.25	1.5^b^ ± 0.15	1.4^b^±0.15	*0.62*	*<0.001*	*0.65*
Σ **n-6 PUFAs**^6^	39.5^a^ ± 1.46	40.1^a^ ± 1.19	49.6^c^ ± 1.80	51.7^c^ ± 1.74	47.0^b^ ± 1.31	***0.022***	***<0.001***	***0.19***
22:5n-3	0.7^b^ ± 0.11	0.7^b^ ± 0.19	0.7^b^ ± 0.13	0.6^ab^ ± 0.09	0.5^a^ ± 0.14	*0.42*	*0.028*	*0.73*
22:6n-3^5^	12.1^c^ ± 1.89	11.9^c^ ± 1.43	7.0^a^ ± 0.48	6.4^a^ ± 0.53	8.5^b^ ± 0.71	*0.21*	*<0.001*	*0.34*
Σ **n-3 PUFAs**^5^	12.8^c^ ± 1.92	12.6^c^ ± 1.43	7.7^ab^ ± 0.46	7.0^a^ ± 0.59	9.0^b^ ± 0.74	***0.17***	***<0.001***	***0.30***
Δ^6^ DS products^7^	90.7^c^ ± 2.39	91.4^c^ ± 2.15	79.1^a^ ± 1.74	78.9^a^ ± 1.61	84.5^b^ ± 1.62	*0.75*	*<0.001*	*0.52*
Ratio n-6/n-3	3.2^a^ ± 0.64	3.2^a^ ± 0.36	6.5^c^ ± 0.54	7.4^d^ ± 0.59	5.3^b^ ± 0.45	*0.017*	*<0.001*	*0.029*
Relative UI	2.29^a^ ± 0.09	2.30^a^ ± 0.09	2.22^a^ ± 0.06	2.26^a^ ± 0.09	2.26^a^ ± 0.07	*0.41*	*0.067*	*0.64*

^1^See footnote 1 of Table 2.

^2^Values are means ± SD, *n* = 8. Labeled means in a row without a common letter differ by 1-factor ANOVA followed by Tukey's multiple-comparison test, *P* < 0.05.

^3^Bifactorial ANOVA of the LZ-CB, HZ-CBR, LZ-SF, and HZ-SFR diet groups.

^4^Contain 16:1, 20:1n-9, and 22:1n-9.

^5^1-factor and bifactorial ANOVA after logarithmic transformation of the data.

^6^Contain 20:2n-6.

^7^Total Δ^6^ desaturation products (mol per 100 mol PUFAs) contain 20:3n-6, 20:4n-6, 22:4n-6, 22:5n-3, and 22:n-3.

SFAs, saturated fatty acids; MUFAs, monounsaturated fatty acids; PUFAs, polyunsaturated fatty acids; UI, relative unsaturation index.

**Table 5:** Fatty acid composition (mol/100 mol) of liver triacylglycerols in male weanling rats fed high-fat diets differing in Zn content and fat source free choice or in restricted amounts for 4 wk.

Fatty acids	Diet groups^1,2^					2 × 2 ANOVA^3^: *P *level		
	LZ-CB	HZ-CBR	LZ-SF	HZ-SFR	HZ-SF	Zn	Fat	Zn × fat
16:0^4,5^	24.7^b^ ± 0.57	28.2^c^ ± 1.68	9.8^a^ ± 0.51	10.3^a^ ± 0.47	10.1^a^ ± 1.21	*<0.001*	*<0.001*	*0.018*
18:0^4,5^	9.5^b^ ± 1.08	8.4^b^ ± 0.97	2.5^a^ ± 0.26	2.3^a^ ± 0.21	2.4^a^ ± 0.24	*0.021*	*<0.001*	*0.48*
Σ **SFAs**^4,5^	34.2^b^ ± 0.82	36.6^b^ ± 1.68	12.3^a^ ± 0.67	12.6^a^ ± 0.58	12.6^a^ ± 1.34	***0.006***	***<0.001***	***0.17***
18:1n-9^4,5^	43.8^c^ ± 2.13	43.5^c^ ± 4.06	8.8^a^ ± 0.67	9.8^ab^ ± 0.83	10.8^b^ ± 1.44	*0.098*	*<0.001*	*0.044*
Σ **MUFAs**^4,5,6^	45.2^c^ ± 2.24	44.7^c^ ± 4.02	9.6^a^ ± 0.59	10.5^ab^ ± 0.87	11.5^b^ ± 1.43	***0.16***	***<0.001***	***0.058***
18:2n-6	14.1^a^ ± 2.00	12.9^a^ ± 2.68	56.6^b^ ± 2.51	56.4^b^ ± 1.68	57.0^b^ ± 1.29	*0.41*	*<0.001*	*0.52*
18:3n-6^4,5^	0.4^a^ ± 0.11	0.4^a^ ± 0.13	1.8^b^ ± 0.49	2.0^b^ ± 0.35	1.5^b^ ± 0.20	*0.72*	*<0.001*	*0.088*
20:4n-6^4^	2.9^a^ ± 0.53	2.3^a^ ± 0.75	11.2^b^ ± 1.47	10.3^b^ ± 1.23	10.5^b^ ± 2.08	*0.051*	*<0.001*	*0.70*
22:4n-6^4^	0.6^a^ ± 0.20	0.7^a^ ± 0.23	4.4^c^ ± 0.41	3.9^bc^ ± 0.68	2.9^b^ ± 0.67	*0.088*	*<0.001*	*0.056*
Σ **n-6 PUFAs**^7^	18.1^a^ ± 2.27	16.3^a^ ± 3.62	76.6^b^ ± 0.91	75.5^b^ ± 1.10	74.6^b^ ± 2.53	***0.072***	***<0.001***	***0.65***
18:3n-3^4,5^	0.5^a^ ± 0.11	0.5^a^ ± 0.14	0.4^a^ ± 0.06	0.4^a^ ± 0.03	0.5^a^ ± 0.06	*0.70*	*0.036*	*0.81*
22:5n-3^4,5^	1.2^c^ ± 0.28	1.4^c^ ± 0.37	0.6^b^ ± 0.07	0.6^b^ ± 0.10	0.3^a^ ± 0.05	*0.90*	*<0.001*	*0.11*
22:6n-3^4,5^	0.8^c^ ± 0.28	0.6^bc^ ± 0.26	0.4^a^ ± 0.06	0.4^a^ ± 0.05	0.5^ab^ ± 0.11	*0.055*	*<0.001*	*0.15*
Σ **n-3 PUFAs**^4,5^	2.5^b^ ± 0.53	2.4^b^ ± 0.46	1.5^a^ ± 0.08	1.4^a^ ± 0.09	1.3^a^ ± 0.15	***0.31***	***<0.001***	***0.77***
Δ^6^ DS products^8^	29.3^c^ ± 2.74	28.3^bc^ ± 1.79	25.8^abc^ ± 3.04	24.8^ab^ ± 2.70	22.9^a^ ± 3.58	*0.28*	*0.001*	*0.96*
Ratio n-6/n-3^5^	7.5^a^ ± 1.77	6.8^a^ ± 1.18	52.8^b^ ± 3.27	55.8^b^ ± 3.35	57.2^b^ ± 5.98	*0.80*	*<0.001*	*0.18*
Relative UI	1.01^a^ ± 0.05	0.95^a^ ± 0.07	2.04^b^ ± 0.04	2.00^b^ ± 0.05	1.97^b^ ± 0.10	*0.011*	*<0.001*	*0.62*

^1^See footnote 1 of Table 2.

^2^Values are means ± SD, *n* = 8. Labeled means in a row without a common letter differ by 1-factor ANOVA followed by Tukey's multiple-comparison test, *P* < 0.05.

^3^Bifactorial ANOVA of the LZ-CB, HZ-CBR, LZ-SF, and HZ-SFR diet groups.

^4^1-factor ANOVA after logarithmic transformation of the data.

^5^Bifactorial ANOVA after logarithmic transformation of the data.

^6^Contain 20:1n-9 and 22:1n-9 (the latter in SF groups only).

^7^Contain 20:2n-6 and 20:3n-6 in SF groups.

^8^Total Δ^6^ desaturation products (mol per 100 mol PUFAs) contain 18:3n-6, 20:3n-6, 20:4n-6, 22:4n-6, 22:5n-3, and 22:6n-3.

SFAs, saturated fatty acids; MUFAs, monounsaturated fatty acids; PUFAs, polyunsaturated fatty acids; UI, relative unsaturation index.

## Abbreviations

CB: Cocoa butter
FA: Fatty acid
HZ: High zinc
LZ: Low zinc
ME: Metabolizable energy
MUFA: Monounsaturated FA
PUFA: Polyunsaturated FA
SF: Safflower oil
PL: Phospholipid
SFA: Saturated FA
TAG: Triacylglycerol
UI: Unsaturation index
Zn: Zinc.
